# Effects of light qualities on the growth phenotype and internal components of *Schizophyllum commune*

**DOI:** 10.3389/fnut.2026.1734280

**Published:** 2026-03-03

**Authors:** Mengyan Zhao, Jinheng Zhang, Xiaoyi Bai, Yunshuang Wang, Xianjin Jin, Shisong Zhang, Sheng Yang, Bing Zhou

**Affiliations:** 1 College of Big Data, Yunnan Agricultural University, Kunming, China; 2 College of Science, Yunnan Agricultural University, Kunming, China

**Keywords:** functional components, growth phenotype, light qualities, nutritional components, *Schizophyllum commune*

## Abstract

**Introduction:**

Light quality is one of the key environmental factors affecting the growth, development, and internal composition of edible fungi. However, its specific effects on *Schizophrenia commune* remain underexplored.

**Methods:**

*S. commune* was cultivated in spectral chambers under eight light quality treatments: dark control (CK), pink (P), green (G), red (R), natural (N), yellow (Y), white (W), and blue (B) light, with a 12 h/12 h photoperiod at 15 µmol·m⁻²·s⁻¹. Phenotypic traits (growth dimensions, biomass, pileus number), color parameters, spectral reflectance, and internal components (amino acids, polysaccharides, protein, cellulose, lipids) were analyzed.

**Results:**

Light quality significantly modulated specific developmental traits. Red light (R) most effectively accelerated early fruiting body formation, while natural light (N) optimized late-stage morphological expansion. Green light (G) uniquely increased the number of leaf-like structures. For quality attributes, natural and blue light enhanced color saturation and visual appeal. Natural and red light improved the flavor profile by increasing the proportion of umami and sweet amino acids. Pink light (P) was identified as optimal for simultaneously boosting the content of polysaccharides, proteins, and cellulose. In contrast, yellow light (Y) and continuous darkness severely suppressed most growth and quality metrics.

**Discussion:**

This study systematically demonstrates the differential regulatory effects of light quality on the phenotype, sensory quality, and nutritional/functional components of *S. commune*. The findings provide a practical, light-quality-based framework for precision cultivation: employing red light for early fruiting, natural light for maturation and coloration, and pink light for enhancing targeted bioactive compounds.

## 1 Introduction

*Schizophyllum commune* (*S. commune*), commonly known as white fungus or white ginseng, belongs to the *Schizophyliaceae* family (1, 2). This edible and medicinal mushroom is prized for its firm texture, delicate aroma, balanced taste, and nutritional richness (3). The fruiting bodies of *S. commune*, which grow in clusters resembling chrysanthemums, are widely distributed in Chinese regions including Yunnan, Guizhou, and Sichuan (4). As a medicinal and edible fungus, *S. commune* possesses substantial nutritional and multiple health benefits, including enhancing immunity, regulating blood sugar and lipids, delaying aging, and exhibiting anti-inflammatory and antibacterial medicinal effects (5).

Due to its considerable medicinal value and antioxidant properties, the cultivation of *S. commune* has been widely adopted and is in high demand. As a form of energy, light influences the metabolism and synthesis of many fungi (6, 7). During the growth of *S. commune*, light serves as a critical factor affecting its development. However, since edible fungi do not perform photosynthesis, their response to light differs from that of green plants. Instead, they perceive light signals through photoreceptors to regulate physiological responses (8, 9).

The color of *S. commune* available on the market is mostly white or grayish-white, with a small portion being light yellow. However, those with darker coloration, tending more towards yellow or light pink, are more popular due to their superior appearance and color quality, and consequently command a higher market value. Several studies have demonstrated that light plays an indispensable role in modulating phenotypic traits, morphological development, and pigmentation during fungal growth. For instance, research by Dong et al. (10) indicated that pink light enhances the yield of *Cordyceps militaris* fruiting bodies and promotes the accumulation of dry matter components such as carotenoids. Furthermore, research by Zhang et al. (11) demonstrated that different light qualities exert varying effects on internal substances such as polysaccharides and proteins within organisms. Additionally, studies by Wang et al. (12) showed that different light conditions also influence the taste and flavor of edible mushrooms. It is evident that light quality significantly affects the morphology, taste, and internal composition of edible fungi.

Compared with traditional light sources such as incandescent lamps and fluorescent lamps, light-emitting diode panels offer advantages including energy efficiency, long lifespan, uniform surface illumination, and high color rendering index. Furthermore, they do not contain fragile components like the tungsten filament found in incandescent lamps or toxic substances such as mercury, making them low-carbon and environmentally friendly. They also enable precise adjustment of intensity and brightness through intelligent control, establishing them as an important supplementary light source for facility edible mushroom production. Currently, research on *S. commune* is relatively limited. In addition, the effects of different light qualities on the phenotype and internal components of *S. commune* have not been reported. Therefore, this study investigated the effects of different light qualities on the growth phenotype and internal composition of *S. commune* and aimed to provide a theoretical basis for the precise induction and regulation of its growth and development.

## 2 Materials and methods

### 2.1 Experimental design

This experiment was conducted in specially designed spectral chambers at the School of Science, Yunnan Agricultural University. A corresponding light panel was fixed above each spectral chamber. There were a total of 8 spectral chambers, one of which served as the dark control group, and the remaining seven were experimental groups corresponding to seven different light qualities: pink light (P), green light (G), red light (R), natural light (N), yellow light (Y), white light (W), and blue light (B). (Eight treatment groups were cultured in eight independent cultivation chambers, respectively. Each treatment group/chamber contained three independent cultivation bags, and each bag bore five fruiting bodies, resulting in a total of 15 fruiting bodies per treatment group/chamber). All fungal sticks used in this experiment were the “Shenzhou 16” space-mutation-bred strains cultivated by the Institute of Botany, Chinese Academy of Sciences. The light/dark (L/D) cycle for each experimental group was 12 h/12 h. The light intensity for each light quality was set at 15 μmol m^−2^ s^−1^. The wavelength peaks of green light, red light, yellow light, and blue light are 525 nm, 660 nm, 585 nm, and 460 nm, respectively. The wavelength of pink light ranges from 400 nm to 760 nm with a peak at 665 nm. Natural light has a wavelength range of 400 nm to 760 nm and a peak wavelength of 583 nm, while white light has a wavelength range of 450 nm to 780 nm and a peak wavelength of 453 nm, measured approximately 10 cm below the light source using a spectroradiometer (HPCS-310X, Hangzhou Hopoo Color Technology Co., Ltd., Hangzhou, China). The temperature inside the spectral chambers was maintained between 20 °C and 25 °C. The relative humidity was 60 ± 5% before fruiting and 90% after fruiting initiation. Purified water was used throughout the growth process.

### 2.2 Sampling and phenotypic measurement

The length, width, and height of *S. commune* after fruiting were measured using a vernier caliper with Bluetooth data output. The dry weight and wet weight of *S. commune* from each spectral chamber after harvesting were measured using a precision electronic balance and a counterbalance scale, and the number of pilei per *S. commune* fruiting body was counted. Five fruiting bodies were randomly sampled from each treatment group, with three replicates per treatment group.

### 2.3 Color parameter determination

The color parameters of *S. commune* were measured using a portable precision colorimeter (WSC-1B, Shanghai Yidian Physical Optical Instrument Co., Ltd., Shanghai, China). The color data were processed to obtain C (color saturation), Hue (hue angle), CCI (color index), and a^*^/b^*^ (color ratio). A higher C value indicates greater color saturation of the fungal species. Hue reflects the coloration of the fungal species. CCI can be used to evaluate changes in the color of the fungal species. a^*^/b^*^ represents the comprehensive color index (13). The calculation formulas are shown as Equations 1–4.


$$\mathrm{Hue}=\tan^{-1}\left(b^{*}/a^{*}\right),\text{if}\;a^{*}>0;\text{and}\;180+\tan^{-1}\left(b^{*}/a^{*}\right),\text{if}\;a^{*}<0$$ (1)

$$C=\sqrt{a^{*2}+b^{*2}}$$ (2)

$$\mathrm{CCI}=1,000\times a^{*}/\left(L^{*}\times b^{*}\right)$$ (3)

$$\mathrm{Color ratio}=a^{*}/b^{*}$$ (4)

### 2.4 Spectral parameters determination

The reflectance of *S. commune* was measured using a field spectroradiometer (IRIS, ASD Inc., Colorado, United States). A reflectance integrating sphere system, comprising the spectroradiometer, a collimated light source, and a white reference panel, was employed to collect the reflectance data from the samples. The measured spectral curves were subsequently analyzed and compiled.

### 2.5 Amino acids determination

This study measured and analyzed some of the amino acids in *S. commune*, totaling 17 types. When determining the types and content of amino acids in *S. commune*, the samples, reagents (such as acetonitrile, etc.; water conforms to GB/T 6682 Grade 1), and standard substance information are first prepared according to specific methods (14), including the preparation of reagents (e.g., 0.1 mol/L phosphate buffer) and standard solutions (stock solutions are prepared via derivation, constant volume adjustment, and filtration; series working solutions are obtained by diluting the stock solution accordingly). Next, the main instruments, such as the liquid chromatograph (Thermo U3000, Thermo Fisher Scientific, Massachusetts, United States) and the high-speed centrifuge (Eppendorf 5702, Eppendorf, Hamburg, Germany), etc. are set up. Subsequently, sample preparation is carried out: 0.1 g of the sample is weighed, hydrolyzed with hydrochloric acid, adjusted to neutral pH, and then centrifuged; the filtrate is derivatized (by adding buffer and DNFB solution for reaction), adjusted to constant volume, and filtered. Finally, under the set chromatographic conditions (mobile phase: acetonitrile, sodium acetate–acetic acid buffer solution, and water; column temperature: 37 °C; flow rate: 1.0 mL/min, etc.; using a specific gradient elution program), the processed samples and standard solutions are analyzed using the liquid chromatograph, thereby completing the amino acid determination.

### 2.6 Polysaccharides determination

When determining the polysaccharide content in *S. commune*, first clarify the required reagents (kits, standards; anhydrous ethanol and concentrated sulfuric acid need to be self-prepared), analytical balances and microplate readers, etc. During the extraction stage, the sample was dried, pulverized, and approximately 0.05 g was weighed, homogenized with water, and extracted in a 100 °C water bath for 2 h. After cooling and centrifugation, the supernatant was collected. A portion of the supernatant was mixed with anhydrous ethanol and left to stand at 4 °C overnight. Following another centrifugation step, the supernatant was discarded, and the precipitate was dissolved in water. For the measurement stage, the microplate reader was preheated and set to 490 nm. Standard solutions at different concentrations were prepared. Then, 200 μL of each standard or sample supernatant was mixed with the reagent and concentrated sulfuric acid. After water bath treatment and cooling, the absorbance was measured, and a standard curve was plotted. The absorbance of the sample was determined following the same procedure. Finally, the sample concentration was calculated based on the standard curve, and the polysaccharide content was computed using the appropriate formula. The calculation formulas are as follows, *n* represents the sample dilution factor, *y* represents the sample concentration obtained from the standard curve, and *W* represents the sample mass. The corresponding formula is provided in Equation 5.


Polysaccharide content=n×y/W (5)

### 2.7 Protein, cellulose and lipids determination

#### 2.7.1 Protein determination

When determining the protein content in *S. commune*, the following should be prepared in advance: reagents (including copper sulfate, potassium sulfate, etc.; methyl red ethanol solution and boric acid solution need to be prepared according to specific methods; water must be GB/T 6682 Grade 1 water), a Shanghai Fiber Inspection KDN-16K automatic Kjeldahl nitrogen analyzer (KDN-16K, Shanghai Fiber Inspection Instruments Co., Ltd., Shanghai, China), an analytical balance, and other related items. During the sample preparation stage, a corresponding weight of solid, semi-solid, or liquid sample was weighed, and copper sulfate, potassium sulfate, and sulfuric acid were added. The mixture was heated until carbonized, then the heating intensity was increased until the liquid turned clear blue-green and transparent, followed by continued heating. After cooling with water, the volume was made up to 100 mL in a volumetric flask. A reagent blank test was conducted simultaneously. For analysis, either the conventional method or the automatic Kjeldahl nitrogen analyzer method could be used (15). In the conventional method, the nitrogen distillation apparatus was set up, boric acid solution and mixed indicator were added to the receiving flask, the sample digest was pipetted into the reaction chamber, and sodium hydroxide solution was added for distillation. The distillate was then titrated with standard sulfuric acid or hydrochloric acid solution until the corresponding endpoint was reached. In the automatic Kjeldahl nitrogen analyzer method, the sample and reagents were added to a digestion tube for digestion, after which the instrument automatically performed liquid addition, distillation, titration, and data recording. Finally, the protein content in the sample was calculated using the calculation formula combined with the specific nitrogen-to-protein conversion factor for the corresponding food. The calculation formulas are as follows, *V*1 represents the volume of sulfuric acid or hydrochloric acid standard titrant consumed by the test solution, *V*2 represents the volume of sulfuric acid or hydrochloric acid standard titrant consumed by the reagent blank, *C* represents the concentration of sulfuric acid or hydrochloric acid standard titrant, *N* represents the mass of nitrogen equivalent to 1.0 mL of 1.000 mol/L sulfuric acid or hydrochloric acid standard titrant, *F* represents the conversion factor from nitrogen to protein, m represents the mass of the test sample, and *V*3 represents the volume of the digested solution aspirated. The corresponding formula is provided in Equation 6.


Protein content=(V1−V2)×C×N×F×100/m×V3/100 (6)

#### 2.7.2 Cellulose determination

When determining the cellulose content in *S. commune*, the following should be prepared in advance: the required reagents (including the assay kit and glucose standard; 80% ethanol, acetone, concentrated sulfuric acid, etc., need to be self-prepared; reagents must be stored as specified; the standard should be prepared as a 10 mg/mL solution before use and then serially two-fold diluted to create a standard curve), a water bath (BJPX-WB26, Shandong Boke Scientific Instruments Co., Ltd., Shandong, China), a microplate reader, and other necessary equipment. During the sample pretreatment stage, take approximately 0.3 g of sample (*m*1), homogenize it with 80% ethanol, incubate in a water bath, and centrifuge. Wash the precipitate with ethanol and acetone to obtain the crude cell wall. Soak the crude cell wall in Reagent 1, then centrifuge, wash, dry, and weigh it (m^2^, representing the CWM mass). Then, weigh approximately 5 mg of CWM (W), homogenize it, add concentrated sulfuric acid, and let it stand in an ice-water bath. After centrifugation, collect the supernatant and dilute it 20-fold for measurement. During the measurement, preheat the microplate reader to 620 nm and set the water bath to 95 °C. Prepare the working solution. According to the loading table, add standard solutions or samples, working solution, and concentrated sulfuric acid into EP tubes. Mix well and incubate in a 95 °C water bath for 10 min. After cooling, measure the absorbance. Plot the standard curve to obtain the regression equation, substitute the sample absorbance to calculate × (mg/mL), and then use the formula to calculate the cellulose content in the sample. The calculation formulas are as follows, *x* represents the glucose concentration obtained from the standard curve, n represents the dilution factor, *V* represents the volume after extraction, *W* represents the mass of CWM used for cellulose extraction, *m*1 represents the total sample mass, and *m*2 represents the mass of the cell wall material (CWM). The corresponding formula is provided in Equation 7.


Cellulose content=x×n×V/W×m2/m1 (7)

#### 2.7.3 Lipid determination

To determine the lipid content in *S. commune*, first specify the required reagents (anhydrous ether, petroleum ether, both of analytical purity, and water meeting GB/T6682 Grade 1 standards), along with the Shanghai Yuejin HH.S21-6-S constant temperature water bath (HH.S21-6-S, Shanghai Yuejin Medical Equipment Co., Ltd., Shanghai, China) and Lichen Technology 101-3bs electric blast drying oven (101-3BS, Lichen Instrument Technology Co., Ltd., Beijing, China). For sample preparation, 2–5 g of solid sample (weighed to 0.001 g precision) was transferred to a filter paper thimble. For liquid or semi-solid samples, 5–10 g (weighed to 0.001 g precision) was placed in an evaporation dish, mixed with quartz sand, evaporated to dryness, dried, ground into fine powder, and then transferred to the filter paper thimble. The dish and glass rod were wiped with ether-soaked degreasing cotton, and the cotton was also placed into the thimble. In the analytical procedure, the thimble was placed into the extraction tube of the Soxhlet extractor, connected to a pre-weighed receiving flask, and anhydrous ethyl ether or petroleum ether was added until it filled two-thirds of the flask’s volume. Reflux extraction was conducted using a water bath at a rate of 6–8 cycles per hour for 6–10 h. Extraction was terminated once the extract showed no oil spots. The solvent was then recovered. When 1–2 mL of solvent remained in the receiving flask, it was evaporated to dryness. The flask was dried at 100 °C ± 5 °C for 1 h, cooled for 0.5 h, and weighed. This process was repeated until a constant weight was achieved (difference between two successive weighings ≤2 mg). Finally, the lipid content in the sample was calculated using the formula. The calculation formulas are as follows, *m*1 represents the mass of the receiving flask and fat after constant weight, *m*0 represents the mass of the receiving flask, and *m*2 represents the mass of the sample. The corresponding formula is provided in Equation 8.


Lipid content=(m1−m0)×100/m2 (8)

### 2.8 Statistical analysis

Spectral data were collected using ViewSpecPro software. Data processing and variance analysis were performed using SPSS Statistics 25 software and Excel software. Graphs were generated using Origin 2024 and Origin 2025b. Orthogonal partial least squares discrimination analysis (OPLS-DA) was conducted with SIMCA 14.1 software. The data are expressed as mean ± standard deviation (SD). A *p*-value <0.05 was considered as statistically significant.

## 3 Results

### 3.1 Analysis of phenotypic traits in *Schizophyllum commune* under different light stresses

**Figure 1:**
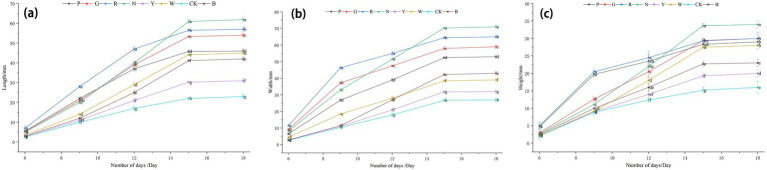
Growth and development of *S. commune* under different light qualities. **(a)** The lengths of *S. commune* fruiting bodies at 6, 9, 12, 15, and 18 days after treatment. **(b)** The widths of *S. commune* fruiting bodies at 6, 9, 12, 15, and 18 days after treatment. **(c)** The heights of *S. commune* fruiting bodies at 6, 9, 12, 15, and 18 days after treatment. Light quality abbreviations: P (pink), G (green), R (red), N (natural), Y (yellow), W (white), CK (dark control), B (blue). Different lowercase letters indicate significant differences between groups (*p* < 0.05) as determined by one-way ANOVA followed by Tukey’s *post-hoc* test.

As shown in Figure 1, among the different light treatments, the growth rate of *S. commune* under red light treatment was the most significant during the early growth stage, as reflected in terms of length, width, and height. During the middle and late growth stages, the growth rates of *S. commune* under red light and natural light treatments were more significant compared to the other treatment groups, with overall levels remaining relatively high. The peak values for length, width, and height of *S. commune* under natural light treatment were 60.97 millimeters, 70.30 millimeters, and 33.68 millimeters (*p* < 0.05), respectively, while under red light treatment, the corresponding peak values were 56.48 millimeters, 64.39 millimeters, and 29.35 millimeters (*p* < 0.05). Therefore, the peak values under red light treatment were slightly lower than those under natural light treatment. Secondly, the growth in length, width, and height of *S. commune* under green light and pink light treatments was relatively lower, with differences in their respective growth rates and peak values. In contrast, the growth of *S. commune* under dark conditions was the slowest in terms of length, width, and height, and throughout the entire observation period, its length, width, and height consistently remained lower than those under other light treatments.

**Figure 2:**
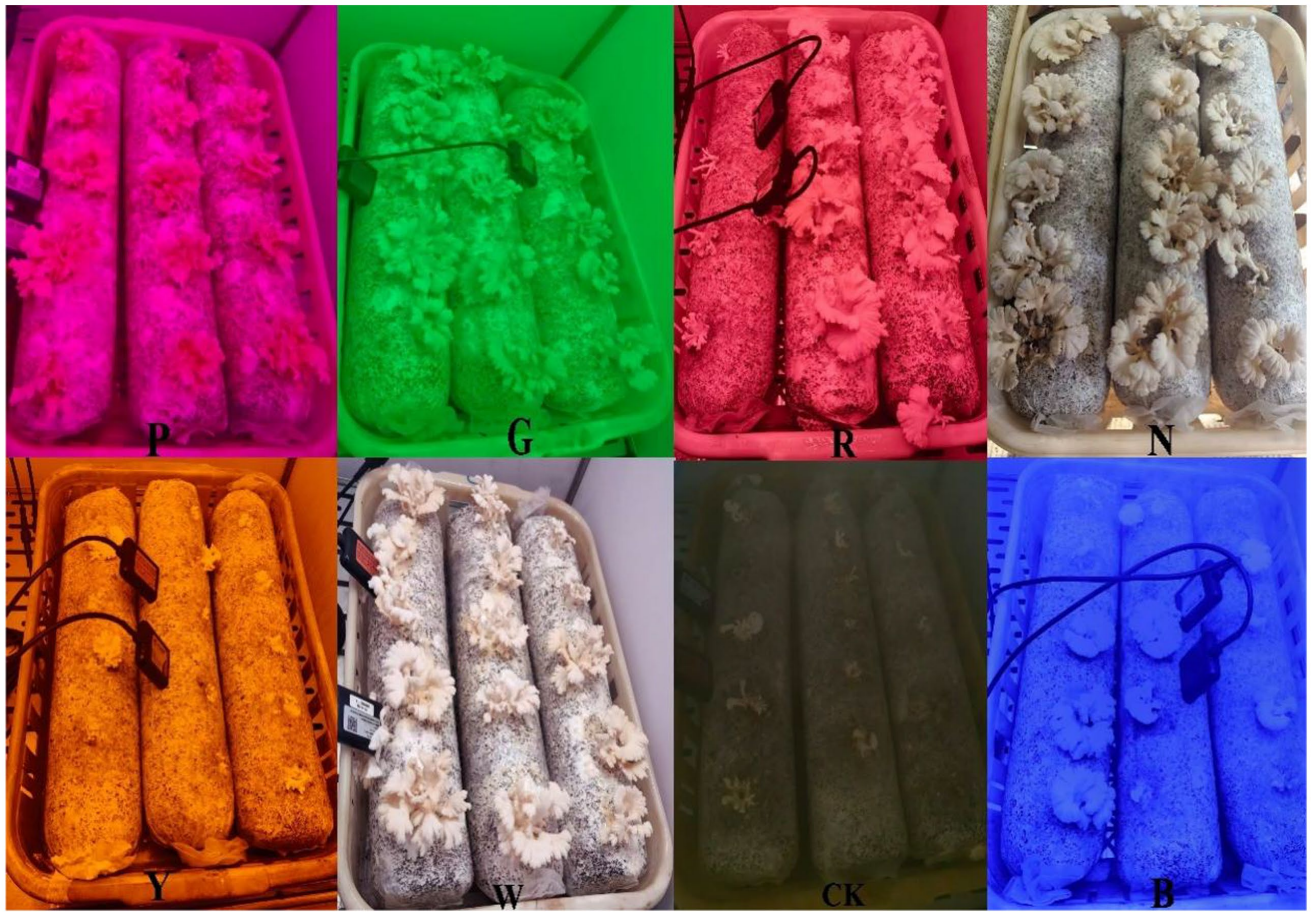
Photos of *S. commune* at the fruiting stage under different light stresses. Images were captured inside the corresponding spectral chambers under the treatment illumination at the defined end-point of cultivation. Each panel shows the complete set of biological replicates for that treatment. Because photographs were acquired under different spectral lighting environments, apparent color/brightness differences across panels reflect illumination conditions.

As shown in Figure 2, overall, the morphological traits of *S. commune* under pink light, green light, red light, and natural light treatments were favorable, exhibiting not only opened but also fan-shaped pileus. With the exception of the dark treatment and yellow light treatment, under which *S. commune* exhibited smaller morphology and relatively closed pileus, the growth under other light qualities was satisfactory. Compared to other light qualities, monochromatic yellow light is unsuitable for cultivating well-developed fruiting bodies (16).

In terms of leaf count, *S. commune* under green light treatment had the highest number of leaves, reaching 217. This was followed by natural light and red light treatments, with 209 and 198 leaves, respectively. Under pink light treatment, the leaf count was relatively high at 110, while white light, blue light, and dark treatments resulted in fewer leaves, with 33, 34, and 26 leaves, respectively. In contrast, yellow light treatment yielded the lowest leaf count, only 18. Overall, the influence of different light qualities on leaf count was quite significant. Green light was more conducive to increasing the number of leaves, whereas yellow light was unfavorable for leaf proliferation.

Regarding biomass under different light qualities, *S. commune* exhibited significant variations in fresh weight (FW), dry weight (DW), and consequently, moisture content (MC). The natural light treatment yielded the highest FW (163.50 g) and MC (83.24%) (*p* < 0.05), indicating that the increased biomass was predominantly due to water uptake rather than solid accumulation. In contrast, the red light treatment, while producing a moderate FW (80.06 g), resulted in the highest DW (38.12 g) and a correspondingly lower MC (52.39%). This suggests that red light is more conducive to the accumulation of dry matter. A similar pattern was observed under green light (FW: 83.10 g; DW: 35.30 g; MC: 57.52%). The lowest biomass values for both FW and DW were consistently recorded under yellow light (5.30 g and 3.60 g, respectively) and dark treatments (5.90 g and 4.11 g, respectively), with their MC also being relatively low (32.11 and 30.36%). Other treatments, such as pink, white, and blue light, resulted in low to moderate FW but were characterized by the lowest MC values (ranging from 24.05 to 44.49%) (*p* < 0.05), highlighting a general trend of efficient water utilization or limited water retention under these specific light conditions.

Overall, the analysis of biomass data (Table 1) indicates that light quality profoundly influences both biomass accumulation and water relations in *S. commune*. Natural light treatment resulted in the highest fresh weight, primarily due to its exceptionally high moisture content (83.24%), suggesting a strong promotive effect on water uptake or retention. In contrast, treatments with red and green light yielded fruiting bodies with a higher proportion of dry matter relative to their fresh weight, indicating a greater accumulation of solid biomass under these conditions. Blue and white light treatments produced fruiting bodies with the lowest moisture content (24.05 and 24.85%, respectively), implying the formation of denser tissue structures. Both yellow light and continuous darkness severely suppressed overall growth, resulting in the lowest absolute values for fresh and dry weight, and also limited water accumulation.

**Table 1:** Weight and moisture content of *S. commune* under different light stresses.

Treatment group	CK	P	G	R	N	Y	W	B
Wet weight	5.90 ± 0.50 g	46.40 ± 0.08d	83.10 ± 0.04b	80.06 ± 0.41c	163.50 ± 0.29a	5.30 ± 0.07 g	11.77 ± 0.14f	13.20 ± 0.16e
Dry weight	4.11 ± 0.04 g	25.76 ± 0.29d	35.30 ± 0.01b	38.12 ± 0.37a	27.40 ± 0.08c	3.60 ± 0.04 h	8.85 ± 0.01f	10.03 ± 0.02e
Moisture content (%)	30.36	44.49	57.52	52.39	83.24	32.11	24.85	24.05

Different lowercase letters indicate significant differences between groups (*p* < 0.05).

### 3.2 Color parameters of *Schizophyllum commune* under different light stresses

This study analyzed the leaf color of *S. commune*, as shown in Table 2. Compared with the control group, *S. commune* under natural light treatment showed a 74.63% increase in the C value, a 1.08% increase in the Hue value, and a 77.78% increase in the a^*^/b^*^ value (*p* < 0.05) Under blue light treatment, the C value increased by 53.80%, the Hue value increased by 0.80%, and the a^*^/b^*^ value increased by 74.10% (*p* < 0.05) The simulated color of *S. commune* under both blue and natural light treatments appeared darker. In contrast, under yellow light treatment, the *C* value decreased by 5.13%, and the simulated color was the lightest. For the other treatment groups, all parameters-*C* value, Hue value, CCI value, and a^*^/b^*^ value-showed an increase compared to the control group. These changes are consistent with the potential enhanced synthesis of dark pigments, such as melanin. Meanwhile, yellow light (Y) led to the lightest coloration and a decrease in color saturation, suggesting an inhibition of such pigment synthesis. The Hue angle, which remained relatively stable across treatments, indicates that the fundamental color character was preserved, but its intensity and shade were modulated.

**Table 2:** Color parameters of *S. commune* under different light stresses.

Treatment group	C	Hue	CCI	a^*^/b^*^	Simulated color
CK	10.13 ± 3.49c	85.02 ± 0.27a	3.96 ± 2.77c	0.27 ± 0.18a	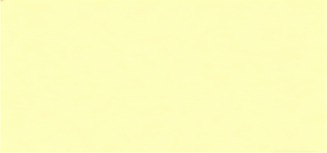
P	11.24 ± 1.92bc	85.27 ± 0.14ab	7.06 ± 2.58bc	0.35 ± 0.10a	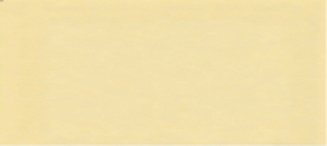
G	12.51 ± 3.18bc	85.30 ± 0.29ab	5.80 ± 3.30bc	0.34 ± 0.17a	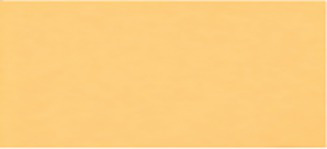
R	13.56 ± 4.16abc	85.57 ± 0.51ab	8.30 ± 5.30abc	0.43 ± 0.24a	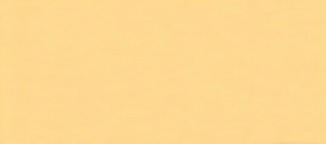
N	17.69 ± 4.87a	85.94 ± 0.67b	11.64 ± 7.04a	0.48 ± 0.25a	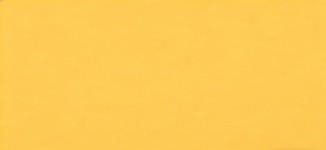
Y	9.61 ± 2.71c	85.10 ± 0.26ab	4.29 ± 2.42c	0.31 ± 0.17a	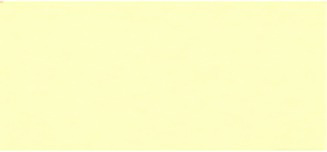
W	12.94 ± 2.90bc	85.57 ± 0.87ab	8.10 ± 6.80abc	0.45 ± 0.33a	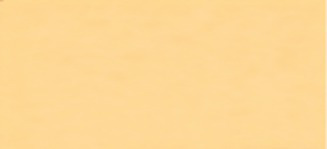
B	15.58 ± 7.72ab	85.70 ± 1.30ab	7.37 ± 6.79ab	0.47 ± 0.46a	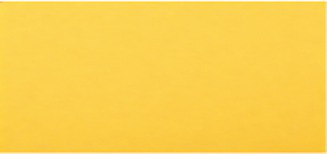

Different lowercase letters indicate significant differences between groups (*p* < 0.05).

Overall, compared with the control, the color of *S. commune* under all light treatments exhibited distinct changes. These results further indicated that blue light and natural light treatments were more conducive to promoting a deeper coloration in *S. commune*, whereas yellow light treatment was unfavorable for enhancing its coloration. This phenomenon may be attributed to light regulation of the synthesis and degradation of specific pigments within the fungal cells. By modulating these key environmental signals, the light qualities are likely perceived by photoreceptor proteins in the fungal cells, thereby activating different pigment synthesis genes and leading to light quality-dependent color variations (17).

From a cultivation perspective, these results provide clear, actionable guidance. Color saturation (*C* value) and the a^*^/b^*^ ratio are robust, non-destructive indicators of pigment development. Monitoring these parameters allows for real-time assessment of fungal physiological status. For cultivators aiming to achieve a deeper, more marketable coloration-often associated with specific phytochemical profiles-the application of blue or natural light is recommended. Conversely, yellow light should be avoided when this is the production goal.

### 3.3 Spectral analysis of *Schizophyllum commune* under different light stresses

**Figure 3:**
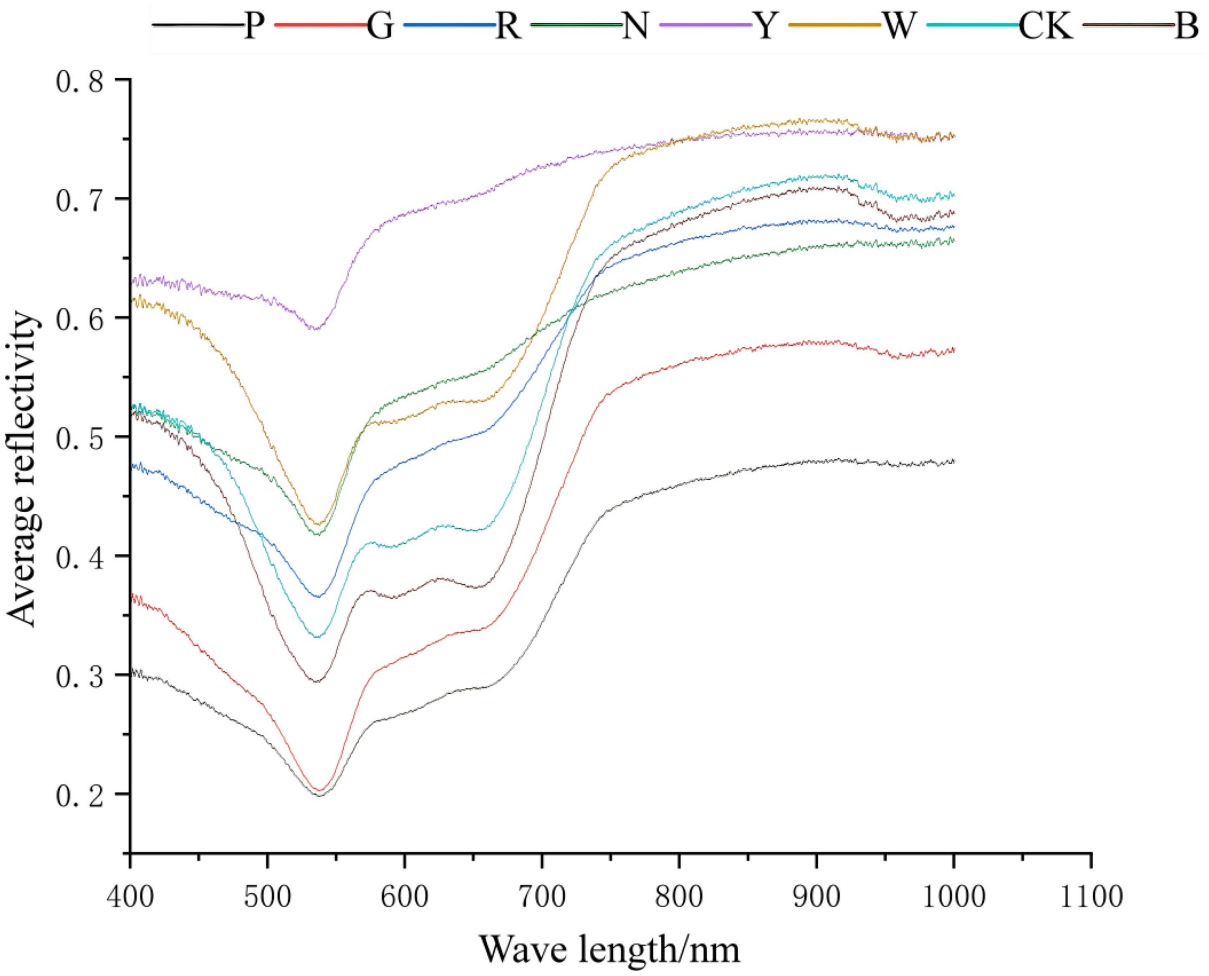
Average reflectance spectra of *S. commune* under different light stresses.

As illustrated in Figure 3, the average reflectance spectra of *S. commune* under various light quality treatments spanned from 400 to 1,000 nm. A consistent reflectance trough was observed around 540 nm, while a prominent peak appeared near 930 nm across all treatments. Notably, the overall reflectance intensity varied significantly among light conditions, the yellow light treatment resulted in the highest reflectance, whereas green and pink light led to the lowest. White, natural, red, blue, and dark treatments exhibited moderate and comparable reflectance levels.

These spectral patterns can be attributed to the selective absorption of light by intracellular components. The light that is heavily absorbed creates reflectance troughs, whereas the unabsorbed light accordingly forms reflectance peaks (18).

The trough at 540 nm aligns with the absorption characteristics of carotenoids—a group of liposoluble pigments, including β-carotene, γ-carotene, and lutein, etc., prevalent in fungi, which absorb strongly in the blue-green region (400–550 nm) (19, 20). The peak at 930 nm may correspond to overtones of O–H and C–H stretching vibrations, often associated with water and organic compounds in fungal tissues.

Furthermore, light stress can trigger defense mechanisms such as cell wall thickening, which enhances light scattering and overall reflectance (21, 22). The elevated reflectance under yellow light may indicate a stress-adaptive response, potentially involving pigment accumulation or structural changes. Conversely, the suppressed reflectance under green and pink light suggests higher absorption or alternative physiological adaptations.

These findings highlight the potential of reflectance spectroscopy as a non-invasive tool for monitoring the physiological status of *S. commune* under different light regimes. Specifically, the reflectance features at 540 nm and 930 nm may serve as indicators of pigment dynamics and structural integrity, offering practical guidance for optimizing light conditions in controlled cultivation environments.

### 3.4 Analysis of amino acids in *Schizophyllum commune* under different light stresses

As shown in Table 3, all 17 amino acids detected in *S. commune* under each light treatment were free amino acids, among which proline (Pro), glutamic (Glu), and arginine (Arg) exhibited the highest contents (ranging from 2.23 to 4.80 mg/100 g), while Lys and Ile showed the lowest contents (ranging from 0.02 to 0.27 mg/100 g).

**Table 3:** Amino acid content of *S. commune* under different light stresses.

Amino acid	Content/(mg/g)							
	CK	P	G	R	N	Y	W	B
Asp	1.40 ± 0.30ab	1.29 ± 0.05bc	1.25 ± 0.15bc	1.16 ± 0.22bc	1.72 ± 0.17a	1.34 ± 0.31bc	2.03 ± 0.04ab	1.02 ± 0.16c
Glu	3.50 ± 0.65b	2.96 ± 0.02bc	3.25 ± 0.49b	2.91 ± 0.61bc	4.80 ± 0.44a	3.17 ± 0.66bc	4.56 ± 0.46bc	2.23 ± 0.43c
Ser	1.60 ± 0.35ab	1.50 ± 0.03ab	1.48 ± 0.17ab	1.35 ± 0.21b	1.89 ± 0.19a	1.55 ± 0.36ab	2.32 ± 0.02ab	1.24 ± 0.18b
Arg	3.24 ± 0.91ab	3.44 ± 0.16ab	3.15 ± 0.59b	2.97 ± 0.50b	4.11 ± 0.27a	3.10 ± 0.57b	4.44 ± 0.17ab	2.48 ± 0.37b
Gly	1.06 ± 0.14ab	0.66 ± 0.03d	0.84 ± 0.05bcd	0.74 ± 0.19 cd	1.24 ± 0.20a	0.98 ± 0.25abc	1.37 ± 0.02abc	0.71 ± 0.12 cd
Thr	2.24 ± 0.48ab	2.05 ± 0.01bc	2.10 ± 0.25bc	1.91 ± 0.27bc	2.71 ± 0.29a	2.14 ± 0.51abc	3.04 ± 0.11abc	1.61 ± 0.26c
Pro	3.79 ± 0.74ab	3.91 ± 0.09ab	3.94 ± 0.29ab	3.45 ± 0.39bc	4.53 ± 0.51a	4.02 ± 0.94ab	4.57 ± 0.24ab	2.82 ± 0.32c
Ala	0.60 ± 0.14ab	0.47 ± 0.01b	0.49 ± 0.07b	0.46 ± 0.04b	0.71 ± 0.09a	0.57 ± 0.16ab	0.73 ± 0.03b	0.44 ± 0.08b
Val	1.39 ± 0.25ab	1.11 ± 0.01bc	1.13 ± 0.13bc	1.10 ± 0.20bc	1.55 ± 0.14a	1.22 ± 0.31abc	1.85 ± 0.08ab	0.98 ± 0.14c
Met	0.40 ± 0.06a	0.37 ± 0.01ab	0.32 ± 0.07abc	0.19 ± 0.12c	0.37 ± 0.08ab	0.29 ± 0.06abc	0.38 ± 0.05abc	0.25 ± 0.09bc
Cys	0.93 ± 0.19a	0.74 ± 0.01ab	0.73 ± 0.08ab	0.68 ± 0.15b	0.95 ± 0.08a	0.81 ± 0.22ab	1.20 ± 0.04ab	0.66 ± 0.10b
Ile	0.12 ± 0.01a	0.05 ± 0.01bcd	0.03 ± 0.01 cd	0.02 ± 0.02d	0.03 ± 0.05 cd	0.04 ± 0.03bcd	0.08 ± 0.01abc	0.08 ± 0.02ab
Leu	0.62 ± 0.14ab	0.56 ± 0.01abc	0.54 ± 0.07bc	0.50 ± 0.09bc	0.74 ± 0.09a	0.58 ± 0.15abc	0.85 ± 0.01abc	0.43 ± 0.09c
Phe	2.36 ± 0.46ab	2.03 ± 0.02b	2.01 ± 0.23b	1.96 ± 0.31b	2.68 ± 0.28a	2.26 ± 0.50ab	3.14 ± 0.09ab	1.84 ± 0.25b
His	0.89 ± 0.21b	0.64 ± 0.05b	0.76 ± 0.13b	0.71 ± 0.24b	1.22 ± 0.13a	0.79 ± 0.25b	1.35 ± 0.08b	0.58 ± 0.13b
Lys	0.08 ± 0.03bc	0.08 ± 0.01abc	0.08 ± 0.02abc	0.07 ± 0.03bc	0.12 ± 0.01a	0.07 ± 0.04bc	0.27 ± 0.01ab	0.05 ± 0.02c
Tyr	0.63 ± 0.24bc	0.67 ± 0.04bc	0.67 ± 0.15bc	0.58 ± 0.21bc	1.07 ± 0.13a	0.64 ± 0.31bc	1.43 ± 0.06ab	0.39 ± 0.13c
Total amino acids (TAA)	24.85 ± 5.22ab	22.52 ± 0.31bc	22.75 ± 2.88bc	20.75 ± 3.65bc	30.44 ± 3.00a	23.56 ± 5.62bc	33.61 ± 1.32abc	17.80 ± 2.80c
Essential amino acids (EAA)	7.21 ± 1.41ab	6.24 ± 0.03bc	6.20 ± 0.75bc	5.74 ± 0.99bc	8.20 ± 0.86a	6.60 ± 1.60abc	9.60 ± 0.25abc	5.24 ± 0.82c
Non-essential amino acids (NEAA)	13.52 ± 2.72b	12.20 ± 0.15bc	12.64 ± 1.42bc	11.32 ± 1.99bc	16.91 ± 1.78a	13.07 ± 3.21bc	18.21 ± 0.87b	9.51 ± 1.51c
Conditionally essential amino acids (CEAA)	4.12 ± 1.11b	4.09 ± 0.13b	3.91 ± 0.71b	3.68 ± 0.72b	5.33 ± 0.38a	3.89 ± 0.82b	5.80 ± 0.24b	3.05 ± 0.49b

Different lowercase letters indicate significant differences between groups (*p* < 0.05).

Among the 17 amino acids identified, threonine (Thr), lysine (Lys), valine (Val), methionine (Met), isoleucine (Ile), leucine (Leu), and phenylalanine (Phe) are essential amino acids (EAA), while aspartic acid (Asp), glutamic acid (Glu), serine (Ser), glycine (Gly), alanine (Ala), proline (Pro), tyrosine (Tyr), and cysteine (Cys) are non-essential amino acids (NEAA). Moreover, histidine (His) and arginine (Arg) are classified as conditionally essential amino acids (CEAA) (13).

**Figure 4:**
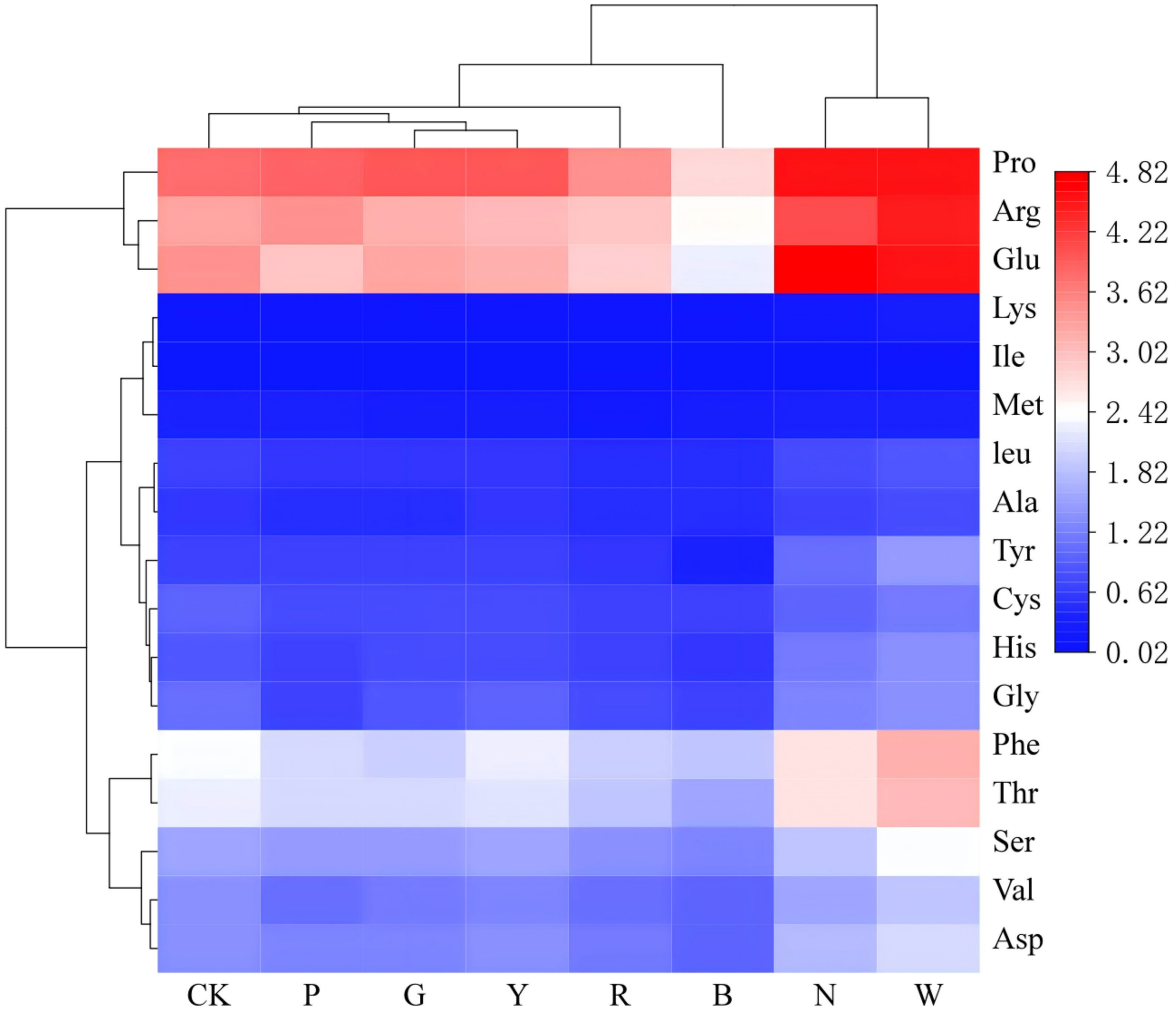
Hierarchical clustering heatmap of amino acid profiles in *S. commune* under different light-quality treatments. Heatmap showing the amino acid composition of *S. commune* cultivated in spectral chambers under eight light conditions: CK (dark control), P (pink), G (green), R (red), N (natural light), Y (yellow), W (white), and B (blue). Seventeen amino acids are displayed (rows), including Pro, Arg, Glu, Lys, Ile, Met, Leu, Ala, Tyr, Cys, His, Gly, Phe, Thr, Ser, Val, and Asp. Amino acids were quantified by liquid chromatography (Thermo U3000, Thermo Fisher Scientific, United States) after derivatization. Color intensity represents amino acid content according to the scale bar [mg/g dry weight (DW)]. Unsupervised hierarchical clustering was performed for both samples and amino acids using Euclidean distance and average linkage. All light treatments were conducted under a 12 h light/12 h dark photoperiod at 15 μmol m^−2^ s^−1^, with chamber temperature maintained at 20–25 °C. Peak wavelengths were: green 525 nm, red 660 nm, yellow 585 nm, blue 460 nm; pink 400–760 nm (peak 665 nm); natural 400–760 nm (peak 583 nm); white 450–780 nm (peak 453 nm).

As shown in Table 3 and Figure 4, compared to the control group, the total amino acid (TAA) content of *Schizophyllum commune* exhibited a declining trend under pink, green, red, yellow, and blue light treatments. In contrast, natural light and white light treatments promoted TAA accumulation. Similarly, the levels of essential amino acids (EAA), non-essential amino acids (NEAA), and conditionally essential amino acids (CEAA) followed a comparable pattern, decreasing under the aforementioned colored light treatments but increasing under natural and white light.

Furthermore, the 17 free amino acids identified in this study comprised 2 umami amino acids (Asp, Glu), 6 sweet amino acids (Thr, Ser, Gly, Ala, Pro, Cys), 8 bitter amino acids (Lys, Val, Met, Ile, Leu, Phe, His, Arg), and 1 tasteless amino acid (Tyr) (13). Compared to the control, the contents of key umami and sweet amino acids (Asp, Glu, Gly, Cys) were elevated under natural and white light, while showing a downward trend under the other light qualities. Notably, the orthogonal partial least squares-discriminant analysis (OPLS-DA) performed later identified five key amino acids with variable importance in projection (VIP) scores greater than 1, which were isoleucine (Ile), cysteine (Cys), methionine (Met), glycine (Gly), and valine (Val) (see section 3.7).

Among these VIP amino acids, the contents of Gly and Cys (sweet amino acids) were significantly elevated under natural light (N) and white light (W) treatments compared to the control (CK). In contrast, the contents of the essential amino acids Ile, Met, and Val were generally suppressed across most light treatments, with pink light (P), green light (G), and red light (R) causing the most substantial reductions.

Regarding the overall taste profile, red light (R) and natural light (N) treatments were conducive to improving flavor, as evidenced by a decreased proportion of bitter amino acids (BAA/TAA) and an increased proportion of umami and sweet amino acids [(UAA + SAA)/TAA] (Table 4). It is worth noting that Val is classified as a bitter amino acid, and its suppression under these light conditions aligns with the observed improvement in flavor profile.

**Table 4:** Content of taste-associated amino acids in *S. commune* under different light stresses.

Amino acid	Content/(mg/g)							
	CK	P	G	R	N	Y	W	B
Umami amino acids (UAA)	4.90 ± 0.95b	4.25 ± 0.05bc	4.50 ± 0.64bc	4.07 ± 0.83bc	6.53 ± 0.61a	4.50 ± 0.97bc	6.59 ± 0.50b	3.25 ± 0.59c
Sweet amino acids (SAA)	10.23 ± 2.00ab	9.33 ± 0.11bc	9.57 ± 0.88abc	8.59 ± 1.22bc	12.02 ± 1.36a	10.07 ± 2.43abc	13.23 ± 0.42ab	7.48 ± 1.05c
Bitter amino acids (BAA)	9.09 ± 2.04ab	8.28 ± 0.15b	8.02 ± 1.22b	7.52 ± 1.43b	10.82 ± 0.96a	8.35 ± 1.90b	12.35 ± 0.35ab	6.68 ± 1.06b
Odorless amino acids (OAA)	0.63 ± 0.24bc	0.67 ± 0.04bc	0.67 ± 0.15bc	0.58 ± 0.21bc	1.07 ± 0.13a	0.64 ± 0.31bc	1.43 ± 0.06ab	0.39 ± 0.13c
UAA/TAA	0.20	0.19	0.20	0.20	0.21	0.19	0.20	0.18
SAA/TAA	0.41	0.41	0.42	0.41	0.40	0.43	0.39	0.42
BAA/TAA	0.37	0.37	0.35	0.36	0.36	0.35	0.37	0.38
(UAA + SAA)/TAA	0.61	0.60	0.62	0.61	0.61	0.62	0.59	0.60

Different lowercase letters indicate significant differences between groups (*p* < 0.05).

### 3.5 Analysis of functional components in *Schizophyllum commune* under different light stresses

**Figure 5:**
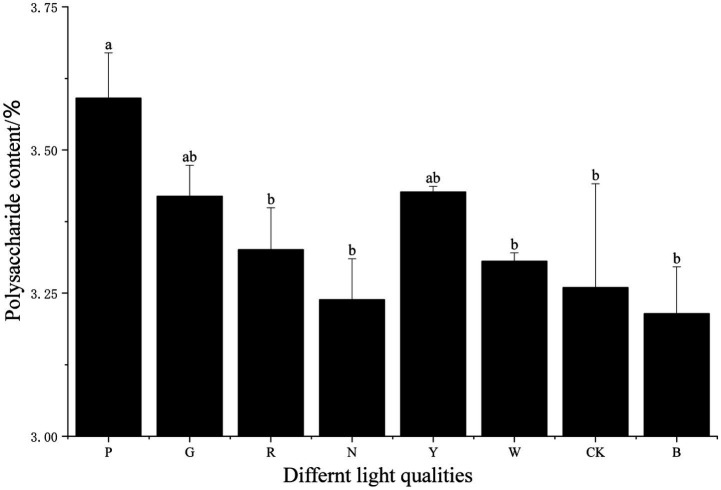
Polysaccharide content of *S. commune* under different light stresses. Polysaccharide content of *S. commune* under eight light conditions: P (pink), G (green), R (red), N (natural light), Y (yellow), W (white), CK (dark control), and B (blue). Polysaccharides were extracted and quantified using the microplate reader method (absorbance at 490 nm) as described in Methods, and content was calculated using Equation 5. Data are presented as mean ± SD. One-way ANOVA followed by Tukey’s HSD multiple-comparison test, Different lowercase letters above bars indicate significant differences among treatments (*p* < 0.05). Experimental conditions: 12 h/12 h L/D, 15 mol m^−2^ s^−1^, 2025 °C.

The functional components of *S. commune* include polysaccharides, phenolic compounds, flavonoids, among others, with primary analysis focused on polysaccharide content. As shown in Figure 5, the polysaccharide content of *S. commune* varied under different light stresses. The highest polysaccharide content was observed under pink light treatment, which increased by 10.15% (*p* < 0.05) compared to the dark treatment. The polysaccharide content under yellow light treatment was the second highest, showing a 5.13% (*p* < 0.05) increase compared to the dark treatment. In contrast, the polysaccharide contents under natural light, white light, blue light, and dark treatments were relatively low. These results indicate that pink light and yellow light treatments are more conducive to the accumulation of polysaccharides in *S. commune*, whereas white light and blue light qualities had the opposite effect.

This might be due to the differential activation of the photoreceptor system and downstream metabolic pathways. Pink light, as a broad-spectrum light with a peak in the red region, might synergistically activate both red- and blue-light sensing pathways, potentially leading to a unique transcriptional reprogramming that favors the allocation of carbon precursors towards polysaccharide biosynthesis (23). On the other hand, the stimulatory effect of yellow light, which is often poorly absorbed by typical fungal photoreceptors, could be an indirect stress response. Sub-optimal light signals might trigger a defense or storage response in the fungus, channeling resources into the synthesis of protective or reserve compounds like polysaccharides (24). Conversely, white light and blue light may prioritize other metabolic processes at the expense of polysaccharide accumulation, explaining its lower yields here (25).

### 3.6 Analysis of nutritional components in *Schizophyllum commune* under different light stresses

The nutritional components of *S. commune* include protein, cellulose, lipids, carbohydrates, etc. The analysis mainly focuses on the protein composition, cellulose composition, and lipid composition.

**Figure 6:**
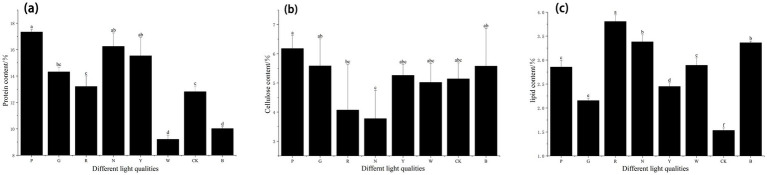
Protein, cellulose, and lipid contents of *S. commune* under different light-quality treatments. **(a)** Protein, **(b)** cellulose, and **(c)** lipid contents of *S. commune* under eight light conditions: CK (dark control), P (pink), G (green), R (red), N (natural light), Y (yellow), W (white), and B (blue). Protein was determined using the Kjeldahl nitrogen method (KDN-16K, Shanghai Fiber Inspection Instruments Co., Ltd., China) and calculated using Equation 6. Cellulose was quantified as described in Methods and calculated using Equation 7. Lipid content was determined by Soxhlet extraction. Data are presented as mean ± SD. Different lowercase letters indicate significant differences (*p* < 0.05). One-way ANOVA followed by Tukey’s HSD multiple-comparison test. Experimental conditions: 12 h/12 h L/D, 15 mol m^−2^ s^−1^, 20–25 °C.

As shown in Figure 6a, significant differences were observed in the protein content of *S. commune* under different light stresses. Among them, the protein content of *S. commune* under pink light treatment was the highest, which increased by 35.25% compared with the dark treatment, followed by that under natural light treatment, which increased by 26.75% (*p* < 0.05) compared with the dark treatment. In contrast, the protein content of *S. commune* under white light treatment was the lowest. Overall, different light stresses significantly affected the protein content, with pink light treatment being more conducive to the increase of protein content in *S. commune*, while white light showed the opposite effect.

As shown in Figure 6b, regarding the cellulose content, the highest cellulose content in *S. commune* was observed under pink light treatment, which increased by 19.87% compared with the dark treatment, followed by that under green light treatment, which increased by 8.63% (*p* < 0.05) compared with the dark treatment. In contrast, the cellulose content of *S. commune* under natural light treatment was the lowest. Overall, different light qualities significantly affected the cellulose content, with pink light treatment being more conducive to the increase of cellulose content in *S. commune*, while natural light showed the opposite effect.

In terms of lipid content, as shown in Figure 6c, there are significant differences in lipid content under different light stresses. Among them, *S. commune* under red light treatment exhibited the highest lipid content, followed by *S. commune* under natural light treatment, while *S. commune* under dark treatment showed the lowest lipid content. Overall, different light stresses had a significant impact on lipid content, with red light being more conducive to increasing lipid content, whereas the dark environment had the opposite effect.

### 3.7 OPLS-DA analysis

**Figure 7:**
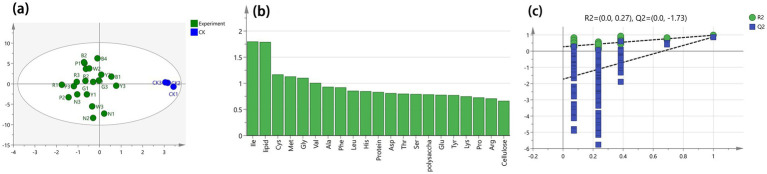
OPLS-DA analysis based on amino acids and major internal components of *S. commune* under different light-quality treatments. **(a)** OPLS-DA score plot showing sample separation under eight light conditions (CK, P, G, R, N, Y, W, B). Each point represents one replicate. **(b)** VIP plot ranking the contributions of amino acids and internal components (polysaccharides, protein, cellulose, and lipids) to group discrimination. **(c)** Model validation output from SIMCA 14.1, showing *R*^2^ and *Q*^2^ values obtained by cross-validation and/or permutation testing.

As shown in Figure 7, OPLS-DA analysis was conducted with 17 free amino acids and 4 internal components as dependent variables and different light stresses as independent variables. The fitting indices for the independent variable (*R*_2_*^x^*), the dependent variable (*R*_2_*^y^*), and the model prediction index (*Q*^2^) in this analysis were 0.998, 0.98, and 0.87, respectively. Both *R*^2^ and *Q*^2^ exceeded 0.5 and were close to 1, indicating a good model fit (26, 27). As shown in Figure 7a, the data points within groups were concentrated, while those between groups were scattered, demonstrating that the experimental methods and instruments were reliable and stable. Therefore, the contents of amino acids and internal components could be used as indicators for sensitivity analysis of *S. commune* to different light stresses. As shown in Figure 7b, a VIP value >1 indicates that the variable contributes significantly to the model and may be a key indicator for analyzing differences (28). Crucially, the variable importance in projection (VIP) plot identified five amino acids with VIP scores greater than 1: isoleucine (Ile), lipid, cysteine (Cys), methionine (Met), glycine (Gly). These compounds are thus the most significant contributors to the metabolic differences observed under various light qualities. Simultaneously, to verify whether the OPLS-DA model was overfitted, resulting in Figure 7c. The results showed that the intersection point of the Q^2^ regression line and the vertical axis was less than 0, and both *R*^2^ (0.27) and *Q*^2^ (−1.73) were below the retention value of 1.0, confirming the absence of overfitting and the validity of the model validation (29).

The prominence of these amino acids suggests a specific metabolic response in *S. commune*. For instance, glutamic and aspartic acids are pivotal umami-tasting amino acids; their variation indicates that light stress can significantly alter the flavor profile of the fungus. Meanwhile, the changes in alanine and arginine may reflect alterations in nitrogen metabolism and energy regulation.

## 4 Discussion

**Figure 8:**
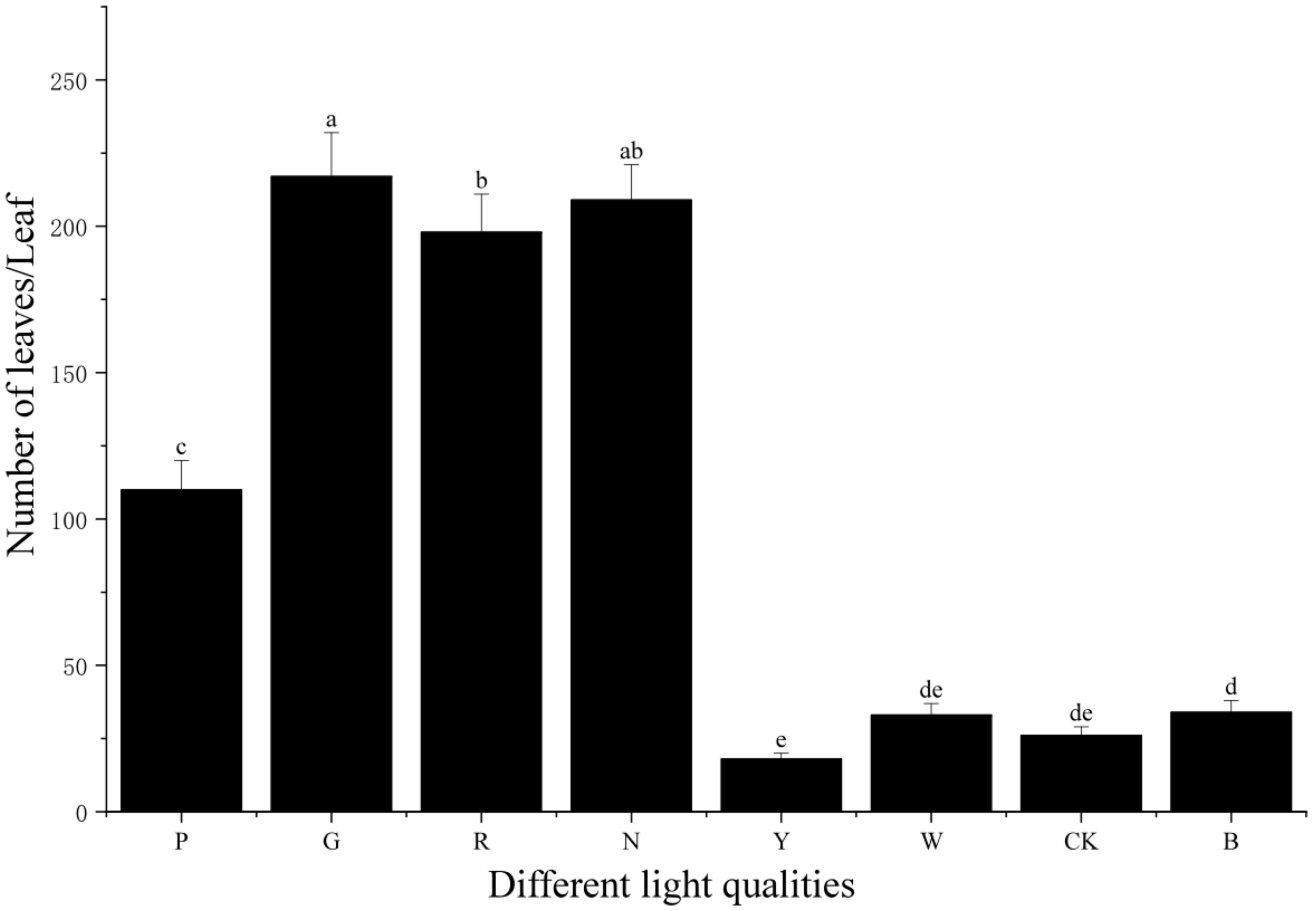
Number of leaves (a) of *S. commune* under different light stresses. Bars represent the mean ± SD. Different lowercase letters indicate significant differences between groups (*p* < 0.05).

This study indicated that light quality significantly influenced the growth phenotype and internal composition of *S. commune* (30). In terms of growth phenotype, our dataset clearly showed the treatment-dependent shifts across three linked outcome domains-biomass at harvest (Table 1), pileus number (Figure 8), and size dynamics (Figure 1), highlighting that “growth” in *S. commune* is multidimensional rather than captured by a single metric. Based on the quantitative endpoints (pileus number, fresh weight, dry weight, and moisture content; Figure 8 and Table 1), compared with the control group, the natural light (N) treatment yielded the highest fresh weight (163.50 ± 0.29 g) and the highest moisture content (83.24%), whereas the red light (R) treatment resulted in the highest dry weight (38.12 ± 0.37 g), followed by green light (G; 35.30 ± 0.01 g). Additionally, natural light substantially increased the moisture content of *S. commune*. Furthermore, green light markedly promoted an increase in the number of fungal pilei (fruiting bodies). By contrast, yellow light (Y) and the dark control (CK) produced the lowest fresh weight (both “g”), and Y also had the lowest dry weight (“h”), indicating strongly suppressed biomass accumulation under these conditions. Together, these patterns suggested that light quality can differentially shape yield quantity (pileus number), biomass allocation (DW vs. FW), and water contribution (moisture content), which should be interpreted as distinct but related aspects of development. These findings align with the research of Yue et al. (31), which indicated that red light and natural light are more conducive to the growth and development of *Pleurotus eryngii*. Studies by Liu et al. (32) and Du et al. (33) also reported that red light favors the growth of fungi, while Liu et al. (9) demonstrated that green light can promote phenotypic traits in *Ganoderma lucidum*. In contrast, blue light treatment reduced both the pileus number and biomass (FW and DW; Table 1) of *S. commune*, which contradicts the findings of Kim et al. (34), who observed that blue light increased the yield of *Lentinula edodes*. This discrepancy may be attributed to differences in metabolic pathways and photoreceptors between the two fungal species and suggested that responses to light quality can be species-dependent. This species-dependence was consistent with the view that fungal photobiology is mediated by distinct repertoires and sensitivities of photoreceptors (e.g., blue-light receptors, red/far-red sensing systems) and downstream regulatory networks, which can lead to divergent developmental outputs across taxa. Notably, standardized phenotypic photographs corresponding to the same biological replicates used for these destructive measurements were not available; therefore, morphology-related interpretations are presented conservatively and are primarily supported by the quantitative data.

The color of mushrooms reflected their freshness and influences consumer purchase intention (30). Compared with the control, *S. commune* with natural light and blue light exhibited deeper and more vivid coloration. Studies by Zhang et al. (35) and Chen et al. (13) have shown that blue light exposure deepens the pigmentation of edible fungi, as their coloration was regulated by pigments such as carotenoids-a process associated with the blue light signaling pathway and coordinated by blue light photoreceptors. In the context of our results, the observed color enhancement under natural and blue light was consistent with prior reports linking blue-light perception to pigmentation pathways; however, due to the pigment composition was not directly quantified in this study, mechanistic inferences should be considered preliminary. From a color perspective, treatments with natural light and blue light are more beneficial for enhancing the appearance of *S. commune*. Regarding spectral reflectance, yellow light treatment increased the average reflectance spectrum of *S. commune* overall compared with the control. This optical signature can be interpreted as a phenotypic readout of tissue-level absorption/scattering properties under yellow illumination. This result may stem from its light absorption characteristics similar to those of plants. Research by Zhang et al. (16) pointed out that plants have low absorption efficiency for yellow light, suggesting that yellow light may likewise be poorly utilized by the photosynthetic pigments or other photoreceptor systems of *S. commune*. Consequently, most of the unabsorbed yellow light is reflected, leading to a higher reflectance spectrum. Because fungi are not photosynthetic, we interpret the plant analogy cautiously; nevertheless, the higher reflectance under yellow light remains a reproducible spectral feature that may relate to wavelength-specific absorption limits and/or structural light scattering, which merits targeted biochemical and microstructural validation in future work.

In terms of flavor and taste, amino acids primarily influence the taste of edible fungi (13). This study found that in *S. commune* treated with red light and natural light, the proportion of bitter amino acids relative to total amino acids decreased, while the proportions of umami and sweet amino acids increased. These compositional shifts provide a plausible basis for altered taste attributes under different light qualities. This may be due to light activating key enzymes that preferentially promote the synthesis of umami and sweet amino acids while inhibiting the synthesis of bitter amino acids. More conservatively, these shifts may reflect wavelength-dependent regulation of nitrogen metabolism and amino-acid interconversion, potentially mediated by photoreceptor signaling and downstream transcriptional control. Simultaneously, light may guide nitrogen sources to prioritize the allocation to high-priority umami and sweet amino acids and adjust cellular structure and transport functions to enhance amino acid accumulation (36, 37). This interpretation was consistent with a broader framework in fungal physiology in which environmental light cues influence carbon-nitrogen balance, resource allocation, and developmental metabolism, thereby reshaping free amino-acid pools.

Regarding functional components, consuming foods rich in polysaccharides can provide antioxidant benefits, enhance immunity, and improve blood glucose and lipid levels. In terms of polysaccharide content, *S. commune* treated with pink light and yellow light exhibited higher polysaccharide levels. Tang et al. (38) found that light affects carbohydrate transport and metabolism in *Lentinula edodes*. It is also possible that light quality, as a wavelength-specific signal, triggers directed gene regulation and metabolic redistribution through the fungal photoreceptor system, altering the activity of key enzymes in polysaccharide synthesis and the flow of carbon sources, thereby disrupting the balance between polysaccharide synthesis and degradation and leading to changes in polysaccharide content (23). In line with these reports, our results suggested that specific light qualities (pink and yellow) were associated with elevated polysaccharide accumulation, supporting the hypothesis that carbohydrate metabolism in fungi is light-responsive. Additionally, studies by Zheng et al. (39), Li et al. (40), and Yeh et al. (24) have shown that yellow light can sometimes be used to enhance certain functions in non-green plants. Although those studies are not fungal systems, they collectively support the general concept that yellow light can act as a biologically meaningful signal in non-photosynthetic contexts; in fungi, such effects would most plausibly be mediated through photoreceptor-dependent signaling rather than photosynthesis.

In terms of nutritional composition, protein, cellulose, and lipids are three major essential nutrients for the human body, promoting digestion, absorption, and maintaining metabolic balance. Nishizawa et al. (41) found that genetic proteins affect the growth and development of fruiting bodies. This study revealed that *S. commune* treated with pink light had the highest protein and cellulose content. Chen et al. (42) discovered that under light influence, some metabolism-related genes are up-regulated, leading to increased protein content. This may also be attributable to the fungal photoreceptor system precisely recognizes specific wavelength light signals and regulates gene expression, metabolic flow, and the balance between protein synthesis and degradation through downstream signaling pathways, resulting in changes in total protein or its composition (43, 44). Furthermore, Ma et al. (45) found that certain enzymes in *S. commune* affect cellulose synthesis. Since light quality acts as a wavelength-specific signal, it triggers directed gene regulation, carbon metabolic redistribution, and enzyme activity adjustments through the fungal photoreceptor system, influencing carbon and enzymes related to cellulose synthesis and degradation, thereby disrupting the balance between cellulose synthesis and degradation (46). *S. commune* treated with red light exhibited the highest lipid content. Research by Turab et al. (47) indicated that the synthesis of fungal lipids is related to the expression of certain genes. Specific light qualities may achieve lipid accumulation by regulating fungal light signal perception, carbon metabolic flow, energy allocation, and stress response, thereby activating lipid synthesis pathways, inhibiting lipid degradation, and optimizing precursor supply (42, 48). Overall, pink light treatment is more conducive to the accumulation of protein and cellulose in *S. commune*, while red light treatment is more favorable for lipid accumulation. Taken together, these component-specific patterns support a targeted interpretation: pink light appears more closely associated with protein/cellulose enrichment, whereas red light is associated with higher lipid levels. Importantly, these associations do not imply single-factor causality; rather, they motivate follow-up mechanistic studies integrating gene expression and enzyme activity assays to map wavelength cues to metabolic outcomes.

The light environment comprises factors such as light quality, light intensity, and photoperiod. This study specifically focused on the effects of light quality on the growth phenotype and internal composition of *S. commune*. The influence of other light-related factors on the growth and development of *S. commune* warrants further investigation. Future work could systematically vary intensity and photoperiod alongside light quality and incorporate standardized phenotypic documentation and pigment/omics measurements to strengthen causal inference and improve the generalizability of cultivation recommendations.

## 5 Conclusion

In conclusion, this study shown that light quality serves as a critical environmental factor for modulating the growth phenotype and internal composition of *S. commune* under the tested conditions. Our results indicate that: (1) For accelerated early development and improved flavor profile, red light is most effective. (2) For maximizing late-stage expansion and visual appeal (color saturation), natural light is advantageous. (3) For targeted enhancement of bioactive compounds (polysaccharides, protein, and cellulose), pink light yields the best results. Conversely, blue light, while beneficial for color deepening, was associated with lower biomass and moisture content. It should be noted that while quantitative data provides valuable insights, the lack of corresponding phenotypic photographic evidence may limit the complete validation of these findings, and future studies could benefit from the inclusion of such visual documentation. Most importantly, conditions of yellow light or continuous darkness failed to promote growth or quality traits, with performance metrics consistently at the lowest levels among all treatments. These findings are synthesized into a practical, light-quality-based framework for *S. commune* cultivation: employing red light in the initial fruiting phase, transitioning to natural light for maturation and coloration, and utilizing pink light when the production goal is to maximize specific functional components. This work provides a foundational guideline for precision management of light regimes to steer the yield, morphology, and nutritional quality of *S. commune*.

## Data Availability

The original contributions presented in the study are included in the article/supplementary material. Further inquiries can be directed to the corresponding author.
